# Longitudinal and Cross-Sectional Analyses of Age Effects on Retinal Nerve Fiber Layer and Ganglion Cell Complex Thickness by Fourier-Domain OCT

**DOI:** 10.1167/tvst.5.2.1

**Published:** 2016-03-04

**Authors:** Xinbo Zhang, Brian A. Francis, Anna Dastiridou, Vikas Chopra, Ou Tan, Rohit Varma, David S. Greenfield, Joel S. Schuman, David Huang, for the Advanced Imaging for Glaucoma Study Group

**Affiliations:** 1 Casey Eye Institute, Oregon Health & Science University, Portland, OR, USA; 2 Doheny Eye Institute, University of California-Los Angeles, Los Angeles, CA, USA; 3 USC Eye Institute, University of Southern California, Los Angeles, CA, USA; 4 Bascom Palmer Eye Institute, University of Miami, Miami, FL, USA; 5 UPMC Eye Center, University of Pittsburgh, Pittsburgh, PA, USA

**Keywords:** nerve fiber layer, ganglion cell complex, thinning, optical coherence tomography (OCT), optic nerve head

## Abstract

**Purpose:**

We studied the effects of age and intraocular pressure (IOP) on retinal nerve fiber layer (NFL) and macular ganglion cell complex (GCC) thickness in normal eyes.

**Methods:**

Data from subjects from the multicenter Advanced Imaging for Glaucoma Study (AIGS) were analyzed. The data included yearly visits from the normal subjects in the AIGS study. Fourier-domain optical coherence tomography (FD-OCT) was used to measure retinal NFL and macular GCC on each visit. Mixed effect models were used to evaluate the longitudinal effect of age and IOP on the NFL and GCC thickness. The measurements at baseline were used to examine the cross-sectional effects.

**Results:**

The analysis included 192 eyes (92 participants) from AIGS between 2009 and 2013. The longitudinal analyses showed overall GCC thickness decreased 0.25 ± 0.05 μm per year (*P* < 0.001) while the overall NFL thickness decreased 0.14 ± 0.07 μm per year (*P* = 0.04). The cross-sectional analyses showed the GCC thickness was 0.17 ± 0.05 μm thinner per year of baseline age (*P* < 0.001), while the NFL was 0.21 ± 0.06 μm thinner (*P* < 0.001). There was no significant IOP effect on either GCC or NFL from either the longitudinal or cross-sectional analysis.

**Conclusions:**

Longitudinal and cross-sectional analyses provided consistent rates of approximately 0.2% per year of age-related thinning in NFL and GCC thicknesses. This is relevant in establishing criteria to detect glaucoma-related thinning (disease progression) in excess of normal aging. IOP does not seem to be a significant confounder for progression analysis.

**Translational Relevance:**

This study demonstrated the relevance of advanced imaging technology in diagnosing and monitoring glaucoma disease.

## Introduction

Optical coherence tomography (OCT) currently is the most commonly used noninvasive imaging technique of the posterior pole of the eye.^1^ With Fournier domain OCT (FD-OCT) technology showing improved resolution and a higher scan rate compared to time domain OCT, it now is possible to evaluate qualitatively and also quantitatively the anatomy of different layers in the human retina and optic nerve head (ONH) in a precise, objective, and reproducible way.^2,3^

Peripapillary retinal nerve fiber layer (NFL) and macula inner retinal layer/ganglion cell complex (GCC) thinning have been recognized as biomarkers of optic nerve damage, especially in glaucoma where the disease is characterized by accelerated retinal ganglion cell (RGC) loss.^4–6^ In the macula, glaucoma affects the inner retinal layers, including the nerve fiber, ganglion cell, and inner plexiform layers, which contain the axons, cell bodies, and dendrites of the ganglion cells. Macular GCC measurements include the thickness of the inner retina extending from the internal limiting membrane (ILM) to the inner plexiform layer. The rationale behind the use of this metric is that the macular region contains a high concentration of more than 50% of RGCs and, thus, could help localize glaucomatous damage or aid in earlier detection.

The role of GCC measurements in the diagnosis and follow-up of glaucoma patients recently has been highlighted in an increasing body of literature.^5–7^ Macular GCC thickness and peripapillary NFL thickness showed similar diagnostic performance for detecting early, moderate, and severe glaucoma,^8–10^ and when added together can be used to improve diagnostic ability, accuracy, and progression detection.^5,9^ In fact, combining structural measurements of GCC, NFL, and disc variables has been suggested to overall improve glaucoma detection.^10^

For NFL and GCC thickness, it is important for progression analysis to discriminate between disease-related change and age-related decline. Indeed, there is a large body of evidence from OCT imaging studies supporting the aging effects on NFL and GCC.^7,11–22^ Most of these studies are cross-sectional in design, for NFL and GCC), with the exception of the longitudinal studies by Leung et al.^23,24^ It also is essential to differentiate real change from artifactual change due to variability in NFL anatomy caused by fluctuations in intraocular pressure (IOP) over time. Because longitudinal studies follow the same patients over time, they are able to quantify and characterize the causative roles of aging and IOP on NFL and GCC thinning. This is in contrast with cross-sectional studies in which their effects can be obscured by other covariates. Therefore, the purpose of this prospective longitudinal study was to quantify the effects of age and IOP in the peripapillary NFL and macular GCC in the same cohort of healthy individuals.

## Methods

### Participants

Participants who were enrolled in the Advanced Imaging for Glaucoma (AIG) Study, a multisite bioengineering partnership and longitudinal prospective clinical study sponsored by the National Eye Institute (ClinicalTrials.gov identifier: NCT01314326), were recruited in this study. The study design and baseline participant characteristics have been reported previously,^25^ and the Manual of Procedures is available online (available in the public domain at www.AIGStudy.net). Clinical data for the AIG Study^25^ were collected from three clinical centers, including the Doheny Eye Institute at the University of Southern California, the University of Pittsburgh Medical Center, and Bascom Palmer Eye Institute at the University of Miami. The study procedures adhered to the Declaration of Helsinki, which guides studies involving human subjects. Written consent was obtained from all participants and proper institutional review board approvals were obtained from all participating institutions.

In this study, data collected from the normal participants from the AIG study were analyzed. Both eyes of the normal participants must meet the following criteria: (1) no history of evidence of retinal pathology or glaucoma, (2) no history of keratorefractive surgery, (3) visual field (VF) tests (mean deviation and pattern standard deviation, and glaucoma hemifield test must be within normal limit, (4) IOP < 21 mm Hg, and (5) must be free of optic nerve damage through slit-lamp biomicroscopy. Normal participants usually provided both eyes for the study and had a yearly follow-up visit after the baseline visit. Since we are evaluating longitudinal change over time, to enter the analysis subjects must have had at least two completed visits to be eligible for the study, so the minimum follow-up time was 1 year. The average IOP from the three Goldmann applanation tonometry measurements taken on each eye at each visit were used for statistical analysis.

### OCT Imaging

Three anatomic regions (optic disc, peripapillary NFL, and macular GCC) were imaged and measured by FD-OCT (RTVue; Optovue, Inc., Fremont, CA). During each visit, participants had three GCC and ONH scans. The macular GCC scan covered a 7 × 7 mm square area in the macula. Scans were centered 0.75 mm temporal to the fovea to improve the coverage of the temporal macula. The macular GCC thickness was defined as the combination of NFL, GCL, and inner plexiform layer.^7^ The automated Optovue software derived a 6-mm diameter GCC thickness map centered 0.75 mm temporal to fovea. The ONH scans consisted of concentric (1.3–4.9 mm diameter) scans and radial scans (3.4 mm length) centered on the optic disc and automatically registered with the 3D disc scan to provide the disc margin information. The NFL thickness profile at D = 3.4 mm was resampled on the NFL map recentered according to detected optic disc center. The radial scans were segmented to calculate the three cup-to-disc ratios (CDRs) and optic disc rim area. The RTVue software (version 6.12) was used to provide the overall average macular GCC thickness and the overall average circumpapillary NFL thickness.

Based on the scan quality recommendations by Zhang et al.,^26^ only noncropping ONH scans with a signal strength index (SSI) above 37 and noncropping GCC scans above 42 were selected for analysis. We also added a suggested SSI-based compensation to NFL thickness: for every 10 units increase (or decrease) in SSI, the measured thickness of NFL was compensated by an increase (or decrease) of 0.56 μm.^26^ All NFL thickness measurements were compensated as if all SSI values were set to 60. Finally, measurements in qualified scans in the same visit were averaged.

FD-OCT data before 2009 were excluded in longitudinal analysis. In year 2009, line camera and spectrometer were updated for RTVue OCT used in the AIG Study, which changed signal strength and signal roll-off with depth. Signal roll-off is an exponential falloff of sensitivity with depth due to limited spectral resolution in the spectrometer unit of OCT. This update improved the image quality but also caused the RTVue software to provide slightly different NFL and GCC thickness measurement, which may bias the longitudinal analysis.

All FD-OCT scans were examined by a certified grader masked to the identity and clinical status of the participant. Scans with cropping of relevant anatomic areas, motion artifacts, or segmentation error were excluded. Valid measurements from each visit were averaged. Eyes with pathologies (e.g., epiretinal membrane, edema) that interfered with GCC or NFL measurements were excluded.

### Statistical Analysis

For longitudinal analysis of age effect, we used mixed effect models on the GCC/NFL thickness measurements. The length of follow-up (in years) is the primary fixed effect in the model. Random effects include variation at individual eye level, variation between visits – this variation can be considered reproducibility, and the correlation between two eyes from the same participant. The slope estimate on the fixed effect of length of follow-up represents rate of change over time on GCC/NFL measurements, after adjusting for variations listed as random effects. We grouped the eyes in three age groups based on their age in the exact middle of the follow-up: less than 55 years, 55 to 65 years, and older than 65 years. Stratified analyses were performed on the mid follow-up age groups to compare the rate of change at different stages in life.

To study cross-sectional analysis for age effect, we used linear model of GCC/NFL thickness measurements on the baseline age and the generalized estimating equations method^27^ was used when applicable to adjust for intereye correlation. Quadratic term for age also was included in both models for GCC/NFL to examine higher order effect than linear alone.

We examined IOP effect by using similar mixed effect model of GCC/NFL with follow-up time and IOP as the fixed effects.

All statistical analyses were performed by SAS 9.3 (SAS Institute, Cary, NC). The level of significance was set at *P* < 0.05.

## Results

The AIG Study enrolled 289 eyes from 145 normal subjects, of which 188 normal eyes from 95 subjects had data scanned after year 2009. Approximately 5% of all scans were rejected due to poor signal strength and another 5% were rejected due to cropping effect. Three more normal subjects (six eyes) were excluded further from all analyses for significant epiretinal membrane or edema in the macula. In the end, 182 eyes from 92 subjects were included in the analysis. The demographic and clinical characteristics of the eyes in the study are listed in Table 1. The values with longitudinal changes (e.g., age, GCC, and NFL) were taken at the first visit after the 12/31/2009 data cutoff point, defining the baseline for the study. In the study, there were more females than males (63% vs. 57%) and 1 of 10 subjects were of African origin. These study subjects were divided roughly equally by their age at the mid-point of the follow-up. There were 58 eyes in the “younger than 55 years” and “55 to 65 years” groups, and 66 eyes in the “65 and older age group.” The age of the study subjects ranged from 40 to 75 years and the average follow-up time was 2.5 ± 1.2 years.

**Table 1:**
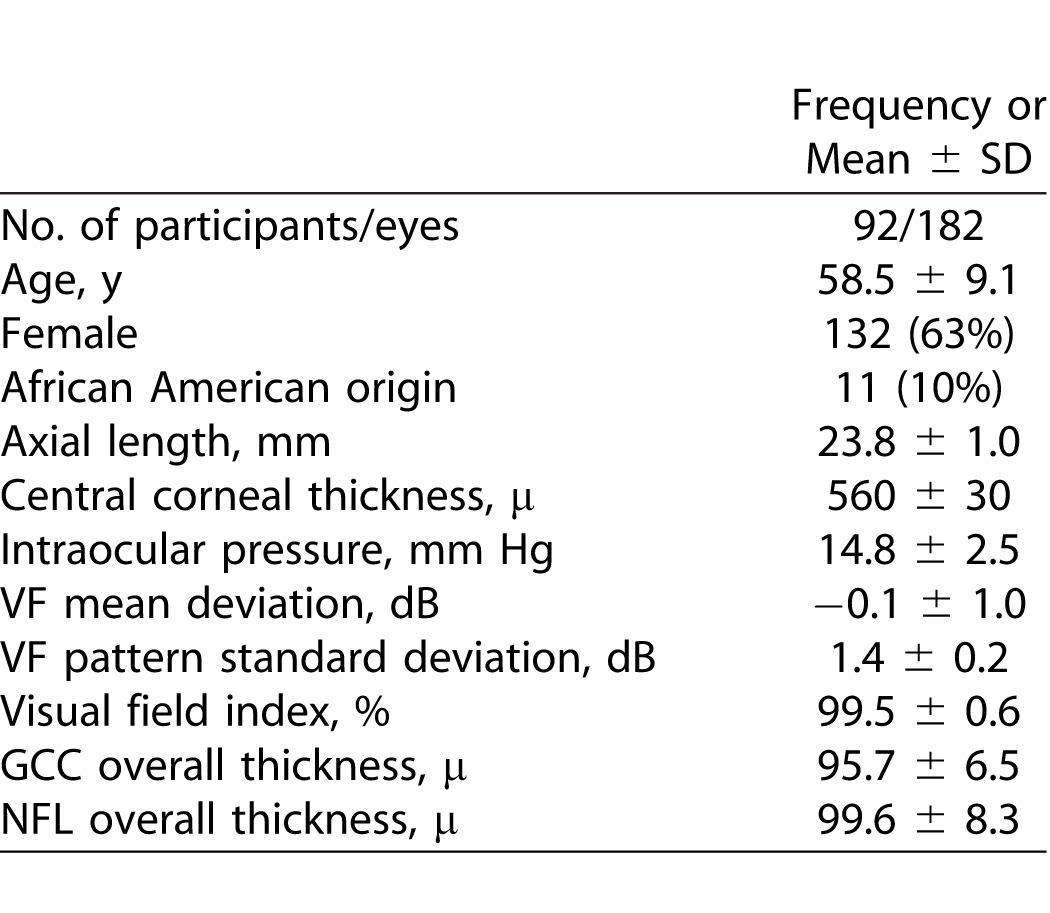
Characteristics of the Study Population at the Baseline Visit

Longitudinal analysis showed the overall GCC thickness significantly decreased at a rate of 0.25 μm/year (95% confidence interval 0.16–0.35 μm/year) averaged across all age groups, which is equivalent to yearly thinning of 0.26% (Table 2). The between-visit reproducibility was 1.39 μm SD. Thus, on an individual basis aging changes would be apparent against measurement noise only after approximately 5 years. GCC decreased faster among the first two younger age groups, and the difference in thinning rate between the 3 age groups was significant (*P* = 0.035).

**Table 2:**
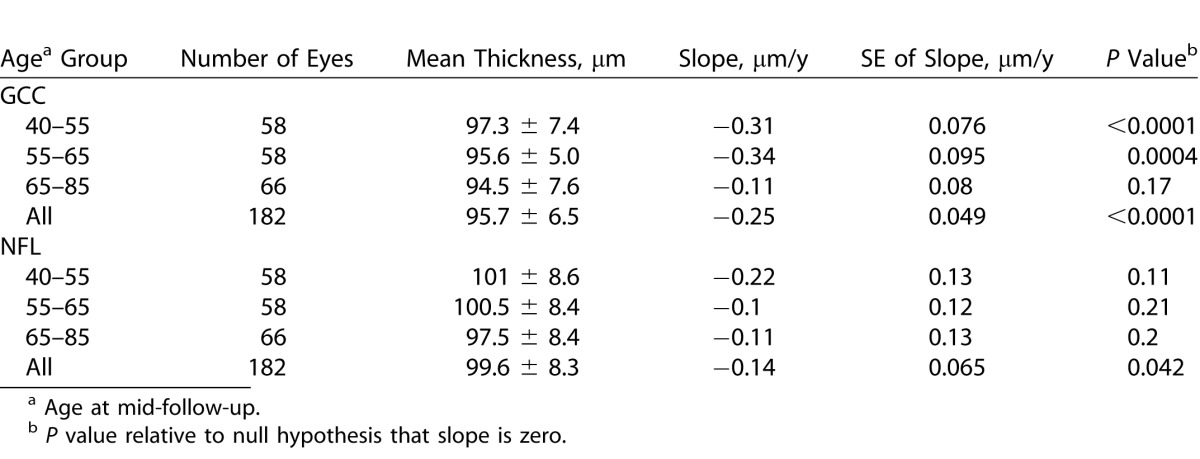
Longitudinal Rate of GCC and NFL Thinning

Longitudinal analysis showed the overall NFL thickness significantly decreased at a rate of 0.14 μm/year (95% confidence interval 0.04–0.26 μm/year) across all age groups (Table 2). The between-visit reproducibility was 2.18 μm SD. Thus, the annual rate of age-related thinning was much smaller than measurement variability, and on an individual basis aging changes would be apparent only after approximately 15 years. The youngest age group appeared to decrease faster than the two older age groups. However, the difference among the 3 age groups was not significant (*P* = 0.44).

**Figure 1:**
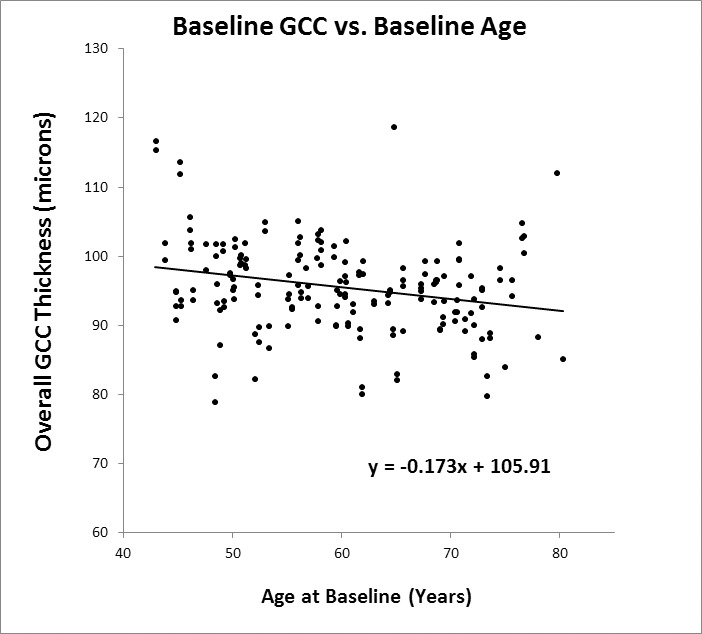
Cross-sectional analysis of macular GCC thickness versus age at the baseline visit. Pearson *R*^2^ = 0.11.

**Figure 2:**
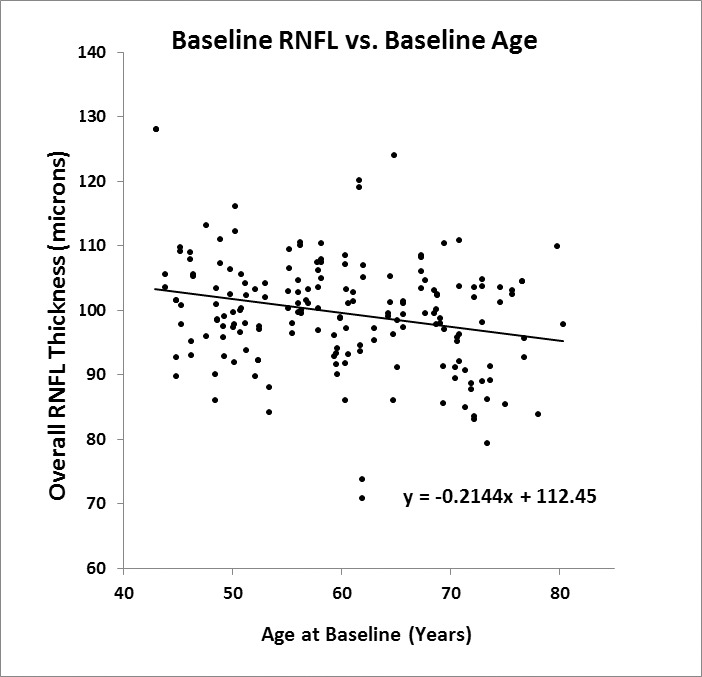
Cross-sectional analysis of peripapillary NFL thickness ersus age at the baseline visit. Pearson *R*^2^ = 0.03.

In the cross-sectional analyses, data from the first visit after 12/31/2009 were used as baseline, and showed that the overall GCC thickness (Fig. 1) was 0.17 μm thinner for each year of age (*P* < 0.001), while the NFL (Fig. 2) was 0.21 μm thinner per year (*P* < 0.001). The slope for GCC was steeper at younger ages (*P* = 0.025 for the quadratic term), which agreed with the faster rate of thinning in the longitudinal analysis. NFL decreased linearly with age (*P* = 0.57 for the quadratic term).

Mixed effects model showed that IOP was not significantly correlated with GCC (*P* = 0.45) nor NFL (*P* = 0.35) thicknesses. The differences in NFL thickness and IOP in consecutive visits were not significantly correlated. The differences in GCC thickness and IOP in consecutive visits also were not significantly correlated. Overall, IOP appeared to have no significant effect on either GCC or NFL measurement.

## Discussion

Characterizing the effects of aging is essential to differentiate between the sequelae of normal aging and progressive thinning due to glaucoma. The rate of detected glaucoma can be affected dramatically when aging changes are taken into account, as shown in a study by Leung et al.^24^ that reported a change in the apparent detection rate of progression based on inner retina thickness from 50% to 20% after adjusting for age. The present study provided quantitative data on the aging effects on NFL and GCC thickness measurements in a large group of healthy subjects aged 40 to 75 years. To our knowledge, this is the largest longitudinal study to evaluate prospectively NFL and GCC changes with age, and the first such study using the RTVue OCT.

Aging already has been shown to affect peripapillary NFL thickness in several cross-sectional studies first with time domain OCT^21^ and then with various SD-OCT systems in individuals with a range of different ethnic backgrounds and in eyes with different axial lengths and refractive errors.^11,13–18,20^ Most studies have found a significant loss in NFL attributed to aging that ranges from 0.15^15^ to 0.56^11^ μm/year, while Rao et al.^17^ did not find a significant effect of aging on NFL thickness in their data. These reports are summarized in Table 3. Several of these studies had large cross-sectional samples (>500 eyes), and they all reported a significant age-related NFL loss. In the Singapore Chinese Eye study, Cheung et al.^19^ reported a decrease of 0.2 μm/year in Chinese aged 40 to 80 years, with NFL imaging performed with the Cirrus HD-OCT (Carl Zeiss Meditec, Inc., Dublin, CA). In the Beijing Eye study, with a larger number of participants, the mean NFL thinning rate with the Spectralis OCT (Heidelberg Engineering, Heidelberg, Germany)^12^ was similar, while the rate of loss was estimated to be larger with the iVue (iVue SD-OCT; Optovue, Inc.).^28^ The effect of aging on the NFL also was investigated with scanning laser polarimetry in the EPIC study in a Caucasian population and a rate of thinning of 0.15 μm/year was reported.^29^ Our cross-sectional results on aging changes and average NFL thickness for normal individuals are in agreement with previous literature.^11–18,20,28^ The advantages of the cross-sectional study design is the ease with which large populations over a wide age range could be measured. However, cross-sectional studies may not provide the cleanest estimate of age-related thinning, since covariates unrelated to aging, such as refractive status, sex, race, smoking history, nutritional and developmental history, and other systemic and eye conditions may vary between the age groups, and these covariates may confound the reported results.

**Table 3:**
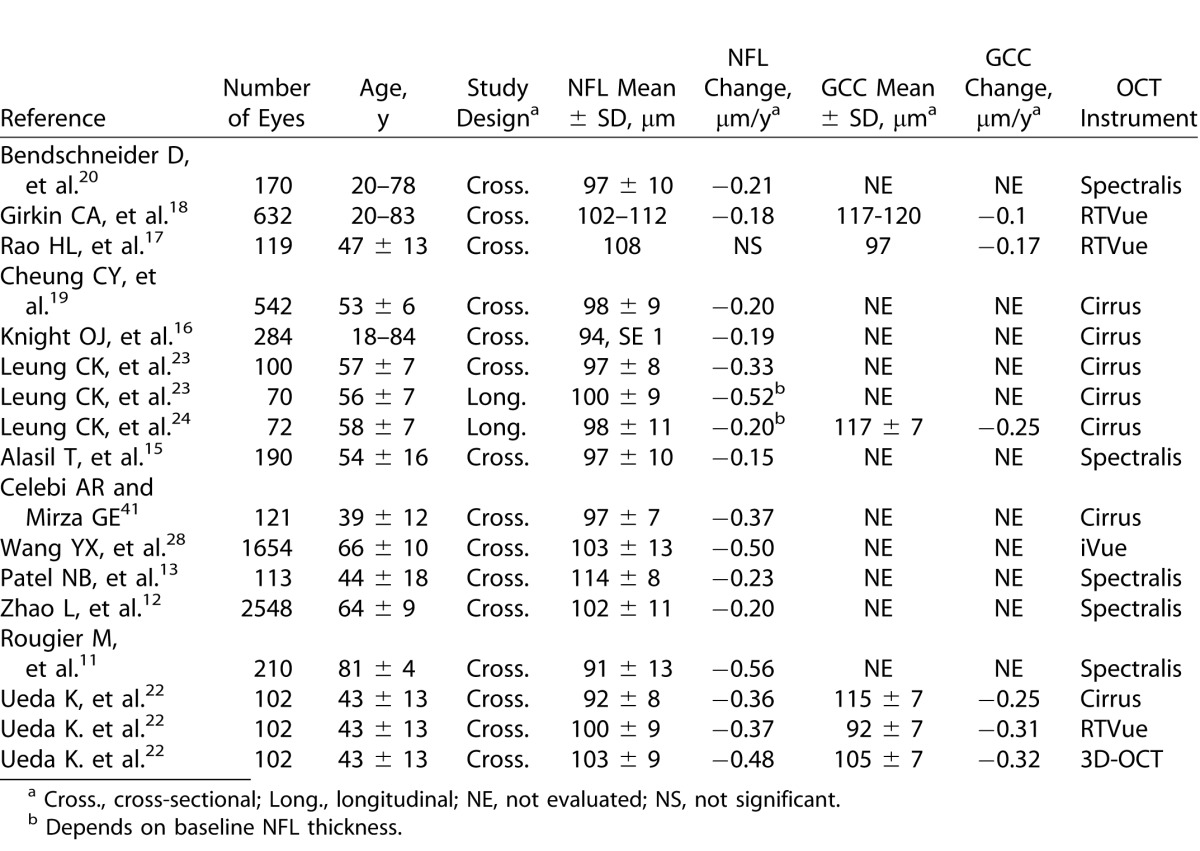
Literature on Aging Effects on NFL and GCC Thickness

Therefore, it is important for these findings to be replicated in longitudinal studies. Leung et al.^23,24^ performed prospective longitudinal studies investigating the effect of aging in NFL measurement with the Cirrus OCT. In a first report in 70 eyes of 35 subjects, they reported a fast rate of NFL thinning of 0.52 μm/year.^23^ In a later article, they reported the rate of thinning as a formula that depended on the baseline thickness in 72 eyes from 40 participants.^24^ The formula yielded a rate of thinning of 0.20 μm/year when their average NFL thickness was entered. Our longitudinal analysis, based on a larger sample of 182 eyes from 92 subjects, resulted in a slower aging rate of 0.14 μm/year. Differences in patient characteristics, sample sizes, and OCT device/software may explain some of the difference. The method of data analysis also may have affected the outcome, as we believe the more complicated statistical model that included baseline thickness may be affected by the regression-towards-the-mean artifact because the baseline thickness was included in the *x* and *y* datasets of their regression analysis.^30,31^ The aging rates of 0.14 and 0.20 μm/year are in statistical agreement. However, the higher rate of 0.52 μm/year reported by Leung et al.^23,24^ may not be realistically fast when extrapolated over decades back to the age of 10 years, which would have yielded a NFL thickness of 124 μm, much thicker than the mean NFL thickness measured in the pediatric age of 98 to 107 μm.^32,33^ The aging rate of 0.14 μm/year when extrapolated back to the age of 10 years, yields an NFL thickness estimate of 106 μm, which agrees better with pediatric results.

Our cross-sectional rate of 0.17 μm/year GCC thinning was within the range of previous literature reports of 0.10 to 0.32 μm/year (Table 1).^17,18,22,24^ In studies with the RTVue, the average GCC thickness decreased by 0.10 to 0.31 μm/year^17,18,22,34^ in different ethnic groups. In other studies with the 3D-OCT (Topcon Corp., Tokyo, Japan) the inner retina thickness would decrease by 0.32 μm every year,^34^ with the ganglion cell layer being thinner by 0.35 μm/year and the inner plexiform layer losing 0.33 μm/year.^14^

To our knowledge, the only previous literature on longitudinally measured GCC aging rate came from Leung et al.,^24^ who reported a decrease of 0.25 μm/year in inner retina thickness measured with the Cirrus, in a normal group of similar age and refractive error, but of different ethnic origin. This agrees with our results within the confidence limits.

In our analysis, we also explored whether the observed rate of NFL and GCC thinning differs depending on the age of the subject. Previous studies have suggested that the rate of thinning may vary with age. Analyzing Spectralis images from an elderly French population, aged 75 years or older, Rougier et al.^11^ reported an accelerated rate of age-related NFL thinning of 0.56 μm/year, which is generally higher than the average observed rate of loss in other studies. On the contrary, our longitudinal analysis of GCC thickness showed that the rate of thinning was much slower in the oldest group with age over 65 years. This was corroborated by our cross-sectional GCC analysis. The 3-fold slowdown of GCC thinning in the oldest group exceeded the slight slowdown one would expect if a fixed percentage of GCC is lost per year rather than a fixed micrometer thickness. Because the slowdown in thinning was not corroborated by our NFL results, we are not confident that the GCC result was not simply due to chance variation. It is not clear why age-related thinning appear to slow down in the oldest participants. While it may be that loss of retinal cells and axons slows down with age, other explanations may be more plausible, for example increase in tissue hydration in the oldest group, or glial proliferation could make the tissue appear thicker, or there may be subtle thickening of the ILM that make the inner retinal appear thicker. There is insufficient data in the study to differentiate between these possibilities. Further studies are needed to assess whether the natural rate of NFL and GCC thinning changes with age. Given our limited sample size, it is more prudent to rely on the simpler linear model for the whole cohort, which yielded the longitudinal rates of −0.25 μm/year (−0.26% per year) for GCC and −0.14 μm/year (−0.14%/year) for NFL. The difference between −0.26% and −0.14% was within the combined standard error of slope measurement. Based on the overall results, the rate of loss of ganglion cells and nerve fibers was approximately −0.2% per year. We have to caution that the *R*^2^ values from Figures 1 and 2 are very low (0.11 for GCC and 0.03 for NFL), suggesting very weak, albeit significant, correlations with age. There also appear to be potential outliers in the plots from Figures 1 and 2, which may have undesired impact on the correlation analyses.

The effect of aging on the population of RGCs has been investigated previously in histology studies, but the findings from different reports have been contradictory. Balazsi et al.^35^ were able to demonstrate an age-related axonal loss in cadaver human eyes. However, this was not replicated in later studies.^36,37^ In fact, Repka and Quigley^36^ found large variability in axonal numbers among their patients which might have obscured the relation with aging and their findings also suggest a redistribution of diameter of the nerve fibers. Jonas et al.^38^ reported an age-related loss in nerve fibers of approximately 5426 nerve fibers/year or 0.5% per year^38^ and Harnan et al.^39^ found a smaller number of neurons in the older compared to the younger retinas. Finally, Gao and Hollyfield^40^ examined the foveal region in eyes from 35 donors and reported that cells in the GCL in the macula decreased by approximately 0.32% per year or by 16% from the second to the sixth decades.^40^ Therefore, there is evidence from histologic studies for an age-related decrease in nerve fiber axons as well as ganglion cells. This generally is in agreement with results from imaging studies with OCT that make it possible to image these changes and objectively quantify them in vivo.

We had analyzed IOP effects due to the theoretical concern that higher IOP could lead to thinner GCC and NFL measurement simply due to elastic stretching of the eye wall. This could be an important effect since IOP is the target of therapeutic interventions for glaucoma and could vary significantly in glaucoma patients when therapy is changed. However, our analysis of the IOP data suggested that in normal eyes, IOP within the normal range does not cause significant variation in NFL or GCC thickness measured by OCT. Although this result does not prove conclusively that IOP could not affect OCT measurements in glaucoma patients, it indicates the effect probably is small relative to overall measurement variability.

In summary, the present study confirmed that there is a significant age-related thinning of NFL and GCC as measured by SD-OCT. The rate of thinning is approximately 0.2% per year based on longitudinal and cross-sectional analyses. The aging change is small relative to intervisit reproducibility; therefore, it does not have a large role in the detection of glaucoma progression over the short term (a few years). However, over many visits and years (e.g., 10 years), the aging effect becomes significant and must be taken into account in the assessment of anatomic damage due to glaucoma.

## References

[1] Huang D, Swanson EA, Lin CP, Optical coherence tomography. *Science*. 1991; 254: 1178–1181. 1957169 10.1126/science.1957169 PMC4638169

[2] Kim JS, Ishikawa H, Sung KR, Retinal nerve fibre layer thickness measurement reproducibility improved with spectral domain optical coherence tomography. *Br J Ophthalmol*. 2009.93: 1057–1063. 19429591 10.1136/bjo.2009.157875 PMC2861342

[3] Leung CK, Cheung CY, Weireb RN, Retinal nerve fiber layer imaging with spectral-domain optical coherence tomography: a variability and diagnostic performance study. *Ophthalmology*. 2009; 116: 1257–1263. 19464061 10.1016/j.ophtha.2009.04.013

[4] Quigley HA, Miller NR, George T. Clinical evaluation of nerve fiber layer atrophy as an indicator of glaucomatous optic nerve damage. *Arch Ophthalmol*. 1980; 98: 1564–1571. 7425916 10.1001/archopht.1980.01020040416003

[5] Tan O, Chopra V, Lu AT, Detection of macular ganglion cell loss in glaucoma by Fourier-domain optical coherence tomography. *Ophthalmology*. 2009; 116: 2305–2314. 19744726 10.1016/j.ophtha.2009.05.025 PMC2787911

[6] Tan O, Li G, Lu AT, Varma R, Huang D. Mapping of macular substructures with optical coherence tomography for glaucoma diagnosis. *Ophthalmology*. 2008; 115: 949–956. 17981334 10.1016/j.ophtha.2007.08.011 PMC2692598

[7] Kim KE, Yoo BW, Jeoung JW, Park KH. Long-term reproducibility of macular ganglion cell analysis in clinically stable glaucoma patients. *Invest Ophthalmol Vis Sci*. 2015; 56: 4857–4864. 25829417 10.1167/iovs.14-16350

[8] Kim NR, Lee S, Seong GJ, Structure-function relationship and diagnostic value of macular ganglion cell complex measurement using Fourier-domain OCT in glaucoma. *Invest Ophthalmol Vis Sci*. 2010; 51: 4646–4651. 20435603 10.1167/iovs.09-5053

[9] Le PV, Tan O, Chopra V, Regional correlation among ganglion cell complex, nerve fiber layer, and visual field loss in glaucoma. *Invest Ophthalmol Vis Sci*. 2013; 54: 4287–4295. 23716631 10.1167/iovs.12-11388 PMC3691052

[10] Loewen NA, Zhang X, Tan O, Combining measurements from three anatomical areas for glaucoma diagnosis using Fourier-domain optical coherence tomography. *Br J Ophthalmol*. 2015; 99: 1224–1229. 25795917 10.1136/bjophthalmol-2014-305907 PMC5457797

[11] Rougier M, Korobelnik JF, Malet F, Retinal nerve fibre layer thickness measured with SD-OCT in a population-based study of French elderly subjects: the Alienor study. *Acta Ophthalmol*. 2015; 93: 539–545. 25586172 10.1111/aos.12658

[12] Zhao L, Wang Y, Chen CX, Xu L, Jonas JB. Retinal nerve fibre layer thickness measured by Spectralis spectral-domain optical coherence tomography: the Beijing Eye Study. *Acta Ophthalmol*. 2014; 92: e35–e41. 23981513 10.1111/aos.12240

[13] Patel NB, Lim M, Gajjar A, Evans KB, Harwerth RS. Age-associated changes in the retinal nerve fiber layer and optic nerve head. *Invest Ophthalmol Vis Sci*. 2014; 55: 5134–5143. 25052998 10.1167/iovs.14-14303 PMC4137486

[14] Demirkaya N, van Dijk HW, van Schuppen SM, Effect of age on individual retinal layer thickness in normal eyes as measured with spectral-domain optical coherence tomography. *Invest Ophthalmol Vis Sci*. 2013; 54: 4934–4940. 23761080 10.1167/iovs.13-11913 PMC5963176

[15] Alasil T, Wang K, Keane PA, Analysis of normal retinal nerve fiber layer thickness by age, sex, and race using spectral domain optical coherence tomography. *J Glaucoma*. 2013; 22: 532–541. 22549477 10.1097/IJG.0b013e318255bb4a

[16] Knight OJ, Girkin CA, Budenz DL, Durbin MK, Feuer WJ. Effect of race age, and axial length on optic nerve head parameters and retinal nerve fiber layer thickness measured by Cirrus HD-OCT. *Arch Ophthalmol*. 2012; 130: 312–318. 22411660 10.1001/archopthalmol.2011.1576 PMC5536837

[17] Rao HL, Kumar AU, Babu JG, Predictors of normal optic nerve head, retinal nerve fiber layer, and macular parameters measured by spectral domain optical coherence tomography. *Invest Ophthalmol Vis Sci*. 2011; 52: 1103–1110. 21087966 10.1167/iovs.10-5997

[18] Girkin CA, McGwin G, Jr, Sinai MJ, Variation in optic nerve and macular structure with age and race with spectral-domain optical coherence tomography. *Ophthalmology*. 2011; 118: 2403–2408. 21907415 10.1016/j.ophtha.2011.06.013

[19] Cheung CY, Chen D, Wong TY, Determinants of quantitative optic nerve measurements using spectral domain optical coherence tomography in a population-based sample of nonglaucomatous subjects. *Invest Ophthalmol Vis Sci*. 2011; 52: 9629–9635. 22039236 10.1167/iovs.11-7481

[20] Bendschneider D, Tornow RP, Horn FK, Retinal nerve fiber layer thickness in normals measured by spectral domain OCT. *J Glaucoma*. 2010; 19: 475–482. 20051888 10.1097/IJG.0b013e3181c4b0c7

[21] Budenz DL, Anderson DR, Varma R, Determinants of normal retinal nerve fiber layer thickness measured by Stratus OCT. *Ophthalmology*. 2007; 114: 1046–1052. 17210181 10.1016/j.ophtha.2006.08.046 PMC2916163

[22] Ueda K, Kanamori A, Akashi A, Effects of axial length and age on circumpapillary retinal nerve fiber layer and inner macular parameters measured by 3 types of SD-OCT instruments [published online ahead of print January 14, 2015] *J Glaucoma*. 10.1097/IJG.0000000000000216 25580890

[23] Leung CK, Yu M, Weinreb RN, Retinal nerve fiber layer imaging with spectral-domain optical coherence tomography: a prospective analysis of age-related loss. *Ophthalmology*. 2012; 119: 731–737. 22264886 10.1016/j.ophtha.2011.10.010

[24] Leung CK, Ye C, Weinreb RN, Yu M, Lai G, Lam DS. Impact of age-related change of retinal nerve fiber layer and macular thicknesses on evaluation of glaucoma progression. *Ophthalmology*. 2013; 120: 2485–2492. 23993360 10.1016/j.ophtha.2013.07.021

[25] Le PV, Zhang X, Francis BA, Advanced imaging for glaucoma study: design, baseline characteristics, and inter-site comparison. *Am J Ophthalmol*. 2015; 159: 393–403.e2. 25447111 10.1016/j.ajo.2014.11.010 PMC4277893

[26] Zhang X, Iverson SM, Tan O, Huang D. Effect of signal intensity on measurement of ganglion cell complex and retinal nerve fiber layer scans in Fourier-domain optical coherence tomography. *Transl Vis Sci Technol*. 2015; 4: 7. 26448900 10.1167/tvst.4.5.7 PMC4594467

[27] Liang KY, Zeger SL. Longitudinal data analysis using generalized linear models. *Biometrika*. 1986; 73.1: 13–22.

[28] Wang YX, Pan Z, Zhao L, You QA, Xu L, Jonas JB. Retinal nerve fiber layer thickness. The Beijing Eye Study 2011. *PLoS One*. 2013; 8: e66763. 23826129 10.1371/journal.pone.0066763 PMC3691254

[29] Khawaja AP, Chan P, Garway-Heath DF, Associations with retinal nerve fiber layer measures in the EPIC-Norfolk Eye Study. *Invest Ophthalmol Vis Sci*. 2013; 54: 5028–5034. 23821204 10.1167/iovs.13-11971 PMC3726240

[30] Chiolero A, Paradis G, Rich B, Hanley JA. Assessing the relationship between the baseline value of a continuous variable and subsequent change over time. *Front Public Health*. 2013; 1: 29. 24350198 10.3389/fpubh.2013.00029 PMC3854983

[31] Stigler SM. Regression towards the mean historically considered. *Stat Methods Med Res*. 1997; 6: 103–114. 9261910 10.1177/096228029700600202

[32] Elia N, Pueyo V, Altemir I, Oros D, Pablo LE. Normal reference ranges of optical coherence tomography parameters in childhood. *Br J Ophthalmol*. 2012; 96: 665–670. 22328811 10.1136/bjophthalmol-2011-300916

[33] Yanni SE, Wang J, Cheng CS, Normative reference ranges for the retinal nerve fiber layer, macula, and retinal layer thicknesses in children. *Am J Ophthalmol*. 2013; 155: 354–360. 23127751 10.1016/j.ajo.2012.08.010 PMC3545013

[34] Kim NR, Kim JH, Lee J, Lee ES, Seong GJ, Kim CY. Determinants of perimacular inner retinal layer thickness in normal eyes measured by Fourier-domain optical coherence tomography. *Invest Ophthalmol Vis Sci*. 2011; 52: 3413–3418. 21357406 10.1167/iovs.10-6648

[35] Balazsi AG, Rootman J, Drance SM, Schulzer M, Douglas GR. The effect of age on the nerve fiber population of the human optic nerve. *Am J Ophthalmol*. 1984; 97: 760–766. 6731540 10.1016/0002-9394(84)90509-9

[36] Repka MX, Quigley HA. The effect of age on normal human optic nerve fiber number and diameter. *Ophthalmology*. 1989; 96: 26–32. 2919049 10.1016/s0161-6420(89)32928-9

[37] Mikelberg FS, Drance SM, Schulzer M, Yidegiligne HM, Weis MM. The normal human optic nerve. Axon count and axon diameter distribution. *Ophthalmology*. 1989; 96: 1325–1328. 2780002 10.1016/s0161-6420(89)32718-7

[38] Jonas JB, Muller-Bergh JA, Schlotzer-Schrehardt UM, Naumann GO. Histomorphometry of the human optic nerve. *Invest Ophthalmol Vis Sci*. 1990; 31: 736–744. 2335441

[39] Harman A, Abrahams B, Moore S, Hoskins R. Neuronal density in the human retinal ganglion cell layer from 16-77 years. *Anat Rec*. 2000; 260: 124–131. 10993949 10.1002/1097-0185(20001001)260:2<124::AID-AR20>3.0.CO;2-D

[40] Gao H, Hollyfield JG. Aging of the human retina. Differential loss of neurons and retinal pigment epithelial cells. *Invest Ophthalmol Vis Sci*. 1992; 33: 1–17. 1730530

[41] Celebi AR, Mirza GE. Age-related change in retinal nerve fiber layer thickness measured with spectral domain optical coherence tomography. *Invest Ophthalmol Vis Sci*. 2013; 54: 8095–8103. 24194190 10.1167/iovs.13-12634

